# Effects of Lateral Size, Thickness, and Stabilizer Concentration on the Cytotoxicity of Defect-Free Graphene Nanosheets: Implications for Biological Applications

**DOI:** 10.1021/acsanm.2c02403

**Published:** 2022-09-12

**Authors:** Chen-Xia Hu, Oliver Read, Yuyoung Shin, Yingxian Chen, Jingjing Wang, Matthew Boyes, Niting Zeng, Adyasha Panigrahi, Kostas Kostarelos, Igor Larrosa, Sandra Vranic, Cinzia Casiraghi

**Affiliations:** † Department of Chemistry, University of Manchester, Oxford Road, Manchester M13 9PL, UK; ‡ Nanomedicine Lab, Faculty of Biology, Medicine and Health, AV Hill Building, University of Manchester, Manchester M13 9PL, UK; § National Graphene Institute, University of Manchester, Booth Street East, Manchester M13 9PL, UK; ∥ Catalan Institute of Nanoscience and Nanotechnology (ICN2), UAB Campus Bellaterra, Barcelona 08193, Spain

**Keywords:** graphene, liquid-phase exfoliation, pyrene, biocompatibility, atomic force microscopy, size-thickness characterization

## Abstract

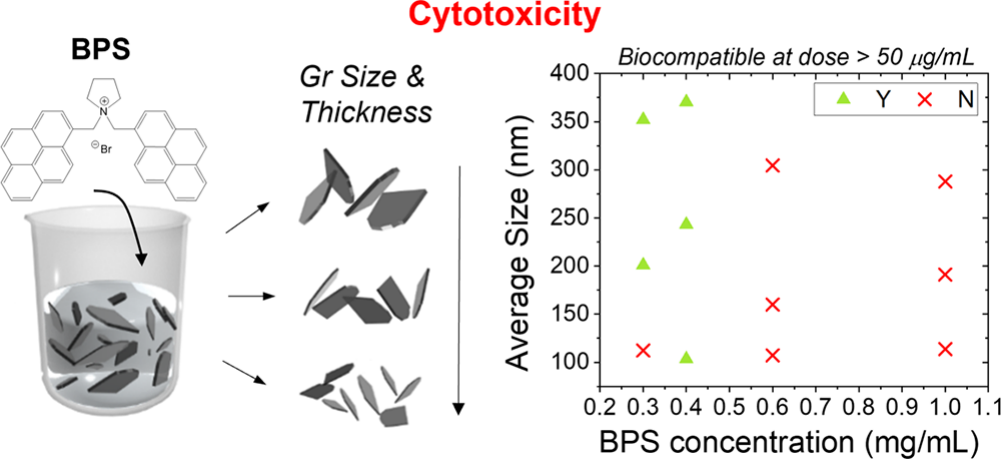

In this work, we apply liquid cascade centrifugation to highly concentrated graphene dispersions produced by liquid-phase exfoliation in water with an insoluble bis-pyrene stabilizer to obtain fractions containing nanosheets with different lateral size distributions. The concentration, stability, size, thickness, and the cytotoxicity profile are studied as a function of the initial stabilizer concentration for each fraction. Our results show that there is a critical initial amount of stabilizer (0.4 mg/mL) above which the dispersions show reduced concentration, stability, and biocompatibility, no matter the lateral size of the flakes.

## 1 Introduction

Two-dimensional (2D) materials have drawn increasing amounts of attention in recent years due to their unique properties.^1−4^ To apply 2D materials in practical applications, low-cost and large-scale synthesis approaches are needed.^4^ Among them, liquid-phase exfoliation (LPE) is an effective and scalable method to produce dispersions of 2D materials.^5,6^ This approach is most commonly performed using organic solvents;^5,6^ however, it can be extended to water with the assistance of suitable stabilizing molecules, enabling us to target biological applications including imaging and drug delivery.^7,8^

Non-covalent functionalization of 2D materials with dispersing agents facilitates the exfoliation and stabilization of 2D materials in water, leading to highly concentrated and stable dispersions.^9−11^ This simple supramolecular approach enables us to tune the exfoliation yield, surface charge, and chemistry by simply selecting different types of dispersants.^12−20^ Among them, small aromatic molecules and, in particular, pyrene derivatives, show very effective exfoliation and stabilization efficiency for graphene in water when compared to conventional surfactants and polymers.^16,21^ However, the exact exfoliation efficiency and dispersion stability strongly depend on the precise structure of the pyrene stabilizer,^14,15,17,18^ whereas the size and thickness of obtained flakes are largely unaffected by the type of pyrene derivative.^13^

**Figure 1:**
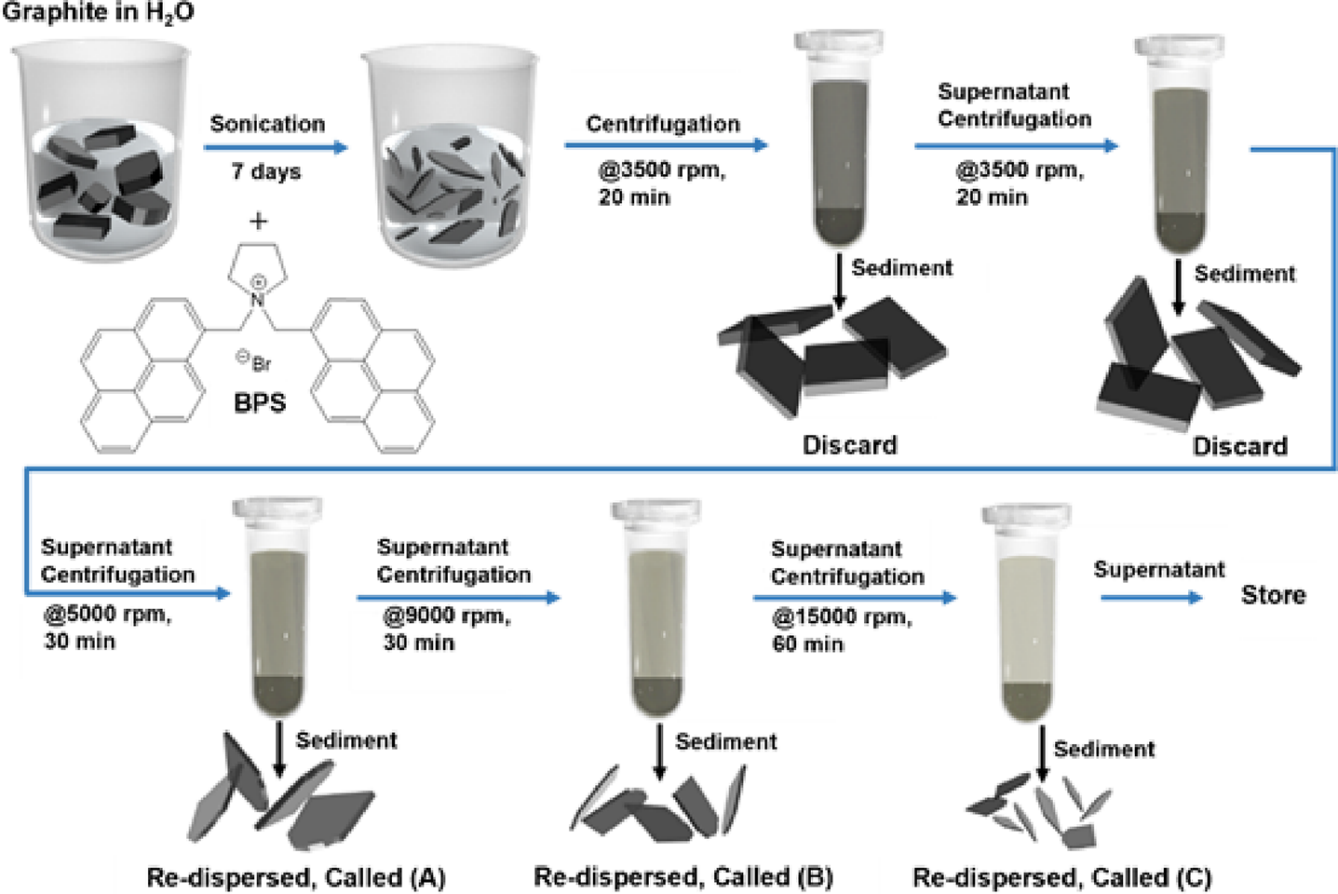
Schematic of the liquid-phase exfoliation of graphite and liquid cascade centrifugation to produce size-selected fractions A, B, and C from a stock dispersion produced with a fixed amount of BPS stabilizer.

Recently, our group has reported the use of a new pyrene derivative, called bis-pyrene (BPS) (Figure 1). The molecular structure of the BPS molecule differs from the one of traditional pyrene derivative stabilizers due to the presence of two pyrene cores functionalized and linked by a single pyrrolidone central group. Despite the molecule being water-insoluble, we observe enhanced exfoliation efficiency in comparison to mono pyrene stabilizers such as 1-pyrenesulfonic acid sodium salt (PS1), which is soluble in water.^14^ This was attributed to the higher interaction strength between BPS and graphene due to the presence of two pyrene groups (rather than just one as in PS1) and the insolubility of the molecule, i.e., the BPS molecule prefers to adsorb onto graphene rather than interacting with water molecules, hence improving the exfoliation efficiency.^14^ When the molecule is adsorbed onto graphene, it prevents reaggregation of the exfoliated nanosheets in solution via electrostatic stabilization, provided by the charged functional group, similar to stabilization with ionic surfactants.^20^ In this work, we exploit the highly concentrated graphene dispersions produced in water using the BPS stabilizer to fraction the nanosheets into narrow size and thickness distributions by liquid cascade centrifugation (LCC).^22^ This enables us to understand how the initial amount of stabilizer and the size and thickness distributions of the graphene nanosheets affect the biocompatibility of these dispersions.

Here, we show a systematic analysis on the cytotoxicity of defect-free graphene dispersions produced by exfoliation in water using BPS. We demonstrate that LCC can be used to fraction the graphene dispersions into stable and concentrated dispersions (concentration > 0.5 mg/mL) with nanosheets with narrow lateral size and thickness distributions, which do not depend on the initial stabilizer amount. Dose-escalation studies performed in the human epithelial bronchial immortalized cell line (BEAS-2B) demonstrated that the cytotoxicity profile strongly depends on the initial amount of stabilizer: below 0.4 mg/mL, the cytotoxicity is affected by the lateral size of the nanosheet, with the largest nanosheets (>200 nm) showing good biocompatibility (up to 75–100 μg/mL dose). Above a 0.4 mg/mL stabilizer concentration, the cytotoxicity does not show any dependence on the lateral size: all fractions show reduced biocompatibility, as compared to the ones obtained with lower stabilizer concentrations.

## 2 Experimental Section

### 2.1 Materials

Graphite flakes (99.5% grade) were purchased from Graphexel Ltd. Silicon wafers (Si/SiO_2_ with an oxide layer thickness ∼290 nm) were purchased from Inseto Ltd. (UK). BPS was synthesized in house as outlined in a previous work.^14^ Deionized water was dispensed from a Millipore Simplicity 185 water purification system. Isopropanol (IPA) was purchased from Sigma-Aldrich.

### 2.2 Preparation of Graphene Dispersions

A Hilsonic bath sonicator (600 W, 30 kHz) with a chiller unit maintaining bath temperature at 10 °C was used for all exfoliation steps. A Sonorex RK 100 bath sonicator (140 W, 35 kHz) was used for substrate cleaning and redispersion of exfoliation material during LCC. A Sigma 1-14k refrigerated centrifuge, with a 12084 rotor and 2.5 mL Eppendorf vials, was used for all centrifugation steps. BPS-stabilized graphene dispersions were produced via LPE in water following the methodology outlined in our previous work.^14^

### 2.3 UV–Vis Spectroscopy

A PerkinElmer l-900 UV–vis–NIR spectrophotometer was used to measure the UV–vis spectrum of graphene dispersions between 250 and 800 nm. Automatic baseline subtraction was used with a DI water baseline. Dilutions of the BPS-stabilized graphene fractions were prepared using DI water, and measurements were taken in UV-cuvettes with a path length of 1 cm. The concentration was calculated by using an absorption coefficient at 660 nm 2460 L g^–1^ m^–1^,^5^ by using the Beer–Lambert law.^23^

### 2.4 Zeta-Potential Measurements

A ZetaSizer Nano ZS purchased from Malvern Instruments, UK was used to obtain the electrophoretic mobility (μ). Dispersions were diluted with DI water and placed in a folded capillary cell for analysis following a default instrument setting at 25 °C and at the natural pH value. In this equipment, zeta-potential (ζ) values were converted from μ by using Henry’s equation.^24^ All samples were measured three times, and the final ζ values were calculated and quoted as mean ± standard deviation (SD).

### 2.5 Dynamic Light Scattering (DLS) Measurements

A ZetaSizer Nano ZS purchased from Malvern Instruments, UK was used to obtain the hydrodynamic size of graphene flakes. Dispersions were diluted with DI water and placed in disposable polystyrene cuvettes (Malvern Instruments, UK) for analysis following a default instrument setting at 25 °C and at the natural pH value. The intensity obtained for each test was used for calculation of Z-average size values after cumulated analysis.^25^ Although these hydrodynamic size values yielded by DLS measurements were not designed for 2D materials, these values can be used to confirm the size evolution during LCC fractionation.^26^ All samples were measured three times, and the mean of the final Z-average values was calculated.

### 2.6 AFM Characterization

A Bruker MultiMode 8 atomic force microscope in PFT with ScanAsyst mode, equipped with ScanAsyst-Air cantilevers, was used for all AFM measurements. Aliquots (10 μL) of each fraction were drop cast onto clean, pre-heated (150 °C), Si/SiO_2_ wafers (0.5 cm^2^), washed with DI water and IPA, and then annealed at 250 °C for 2 h. AFM measurements were taken on each sample, following the methodology outlined in detail in ref (13) and collecting multiple maps capturing over 150 isolated flakes for each fraction. The morphological parameters, lateral size (*L*) and apparent thickness (*T*), were then extracted automatically from the maps using the Gwyddion software, as described in ref (13).

### 2.7 Raman Spectroscopy

A Renishaw Invia Raman spectrometer was employed for Raman measurements. A laser at 514.5 nm with 2.0 mW laser power was used for all measurements. We measured 40–50 isolated flakes, drop cast onto Si/SiO_2_ substrates, for each fraction. A 100× (NA = 0.85) objective lens and a 2400 grooves/mm grating were used. The qualitative thickness distribution of the graphene nanosheets was extracted by fitting the 2D peak of each spectrum with a Lorentzian line shape. The coefficient of determination, *R*^2^, is then used to discriminate between single-layer graphene (SLG) with *N* = 1 (associated to *R*^2^ = 0.99–1.00), few-layer graphene (FLG) with 1 > *N* < 7 (associated to *R*^2^ = 0.97–0.98), and bulk graphite with *N* > 10 (associated to *R*^2^ = 0.95–0.96). A symmetric 2D peak corresponds to SLG, an asymmetric 2D peak corresponds to FLG, and two peaks correspond to bulk graphite. This qualitative method enables us to extract thickness distributions from the Raman spectra, as described in more detail in previous works.^27−31^

### 2.8 Thermogravimetric Analysis (TGA)

TGA experiments were carried out on a TA Instruments SDT-650. Measurements were made in a nitrogen atmosphere with a heating rate of 10 °C/min from room temperature to 800 °C. To isolate the graphene from the dispersions, vacuum filtration was utilized to produce membranes of the material. These membranes were then dried under vacuum at 250 °C and torn for the TGA experiments.

### 2.9 Cell Culture

The human epithelial bronchial immortalized cell line (BEAS-2B, CRL-9609TM, ATCC, and LGC standards, UK) was maintained in an RPMI 1640 cell culture medium (Sigma-Aldrich, Merck Sigma, UK) and supplemented with 10% fetal bovine serum (FBS, Gibco, Thermo Fisher Scientific, UK) and 1% penicillin–streptomycin (Sigma-Aldrich, Merck Sigma, UK) at 37 °C in a humidified 5% CO_2_ incubator. Cells were passaged twice a week using a 0.05% Trypsin–EDTA solution (Sigma-Aldrich, Merck Sigma, UK) when reaching 80% confluence. The activity of trypsin was stopped using 10% FBS.

### 2.10 Cell Treatments

Cells were seeded in 12-well plates (Corning, Costar, Sigma-Aldrich, Merck Sigma, UK) for toxicity assessment performed using optical microscopy and flow cytometry or in CellviewTM cell dishes (627870, Greiner Bio-One Ltd., UK) for uptake experiments, using confocal microscopy. Cells were seeded and treated (when reaching 60–80% confluence) in a complete cell culture medium (see the Cell Culture section), unless stated otherwise.

### 2.11 Optical Imaging and Flow Cytometry

Cells were treated with GR0.3A/B/C, GR0.4A/B/C, GR0.6A/B/C, and GR1.0A/B/C (at graphene concentrations of 25, 50, 75, and 100 μg/mL, 1 mL/well, respectively) in serum-free RPMI for the initial 4 h. After 4 h of incubation, FBS (100 μL/well) was added to the cells and the cells were further incubated for another 20 h. After 20 h of incubation, images were taken using an EVOS FL microscope (10× objective, transmitted channel). GR0.3 and GR0.4 treated cells were detached using Trypsin–EDTA (300 μL/well, 5 min), neutralized with FBS (30 μL/well), centrifuged (1500 rpm, 5 min), resuspended in 1× diluted annexin-binding buffer (200 μL/sample, Molecular Probes, Thermo Fisher Scientific, UK), and stained with annexin V Alexa Fluor 488 (AV) in the dark (1 μL/sample, 20 min, room temperature, Thermo Fisher Scientific, UK). The samples were stored in ice, and propidium iodide (PI, 1 μL/sample, Sigma-Aldrich, Merck Sigma, UK) was added shortly before analysis. A population of 10,000 cells was analyzed on a BD FACSVerseTM flow cytometer with 488 nm excitation. Band-pass filters (515 and 615 nm) were used for annexin V and propidium iodide detection, respectively.

### 2.12 Confocal Microscopy

Cells were treated with GR0.3A/C (graphene concentration = 25 μg/mL, 0.5 mL/well), GR0.6A/C (graphene concentration = 10 μg/mL, 0.5 mL/well), and GR1.0A/C (graphene concentration = 10 μg/mL, 0.5 mL/well) for 24 h. Cells were washed (RPMI culture medium with 10% FBS, 0.5 mL/well, ×2) and incubated in CellTrackerTM Green dye, a green 5-chloromethylfluorescein diacetate (CMFDA) containing solution (3 μM, 0.5 mL/well, 15 min, diluted in RPMI culture medium with 10% FBS, C7025, Thermo Fisher Scientific, UK). After incubation, the CellTrackerTM Green CMFDA dye-containing solution was removed and replaced by the RPMI culture medium with 10% FBS (0.5 mL/well). Cells were then examined under a Zeiss 780 confocal laser scanning microscope using a 40× objective. The confocal images were processed by the Zeiss microscope software ZEN. Excitation/emission wavelength: FDA = 492/517 nm.

## 3 Results and Discussion

### 3.1 Size and Thickness Characterization

In our initial study using the BPS stabilizer,^14^ we have shown that the initial amount of BPS affects the final graphene concentration. In particular, dispersions produced using an initial amount of BPS less than 0.3 mg/mL resulted in the lowest graphene concentrations.^14^ Therefore, for this study, we prepared four graphene dispersions using different initial amounts of BPS equivalent to 0.3, 0.4, 0.6, and 1.0 mg/mL water solvent. The corresponding samples were named GR0.3, GR0.4, GR0.6, and GR1.0, respectively. In detail, graphite (3 mg/mL), BPS (0.3, 0.4, 0.6, and 1.0 mg/mL), and DI water (100 mL) were added to a reagent bottle (250 mL) and sonicated for 7 days continuously. The unexfoliated graphite was removed by centrifugation at 3500 rpm (903 g) for 20 min, which was repeated twice. This step was used to sediment a large amount of the residual unexfoliated graphite from the stock dispersion, which was then discarded, while the supernatant was retained. LCC was then used to separate the resulting supernatant into fractions containing flakes with differing distributions in lateral size and thickness. The following cascade was used for each dispersion: 5000 rpm (1844 g) for 30 min, 9000 rpm (5976 g) for 30 min, and 15,000 rpm (16,603 g) for 60 min. For each dispersion, prepared with a fixed amount of BPS, we obtained three sub-sets of samples, denoted with A, B, and C, e.g., GR0.3A, GR0.3B, and GR0.3C, respectively. Figure 1 shows a schematic of the LPE of graphite and the following LCC methodology that was employed in this work. LCC is expected to result in the sedimentation of heavier and larger flakes at lower centrifugal forces and smaller and thinner flakes at higher centrifugal forces.^22^ Each fraction should therefore contain flakes of a range of sizes and thicknesses that sediment between the higher and lower centrifugal forces used in each step of the cascade.

**Figure 2:**
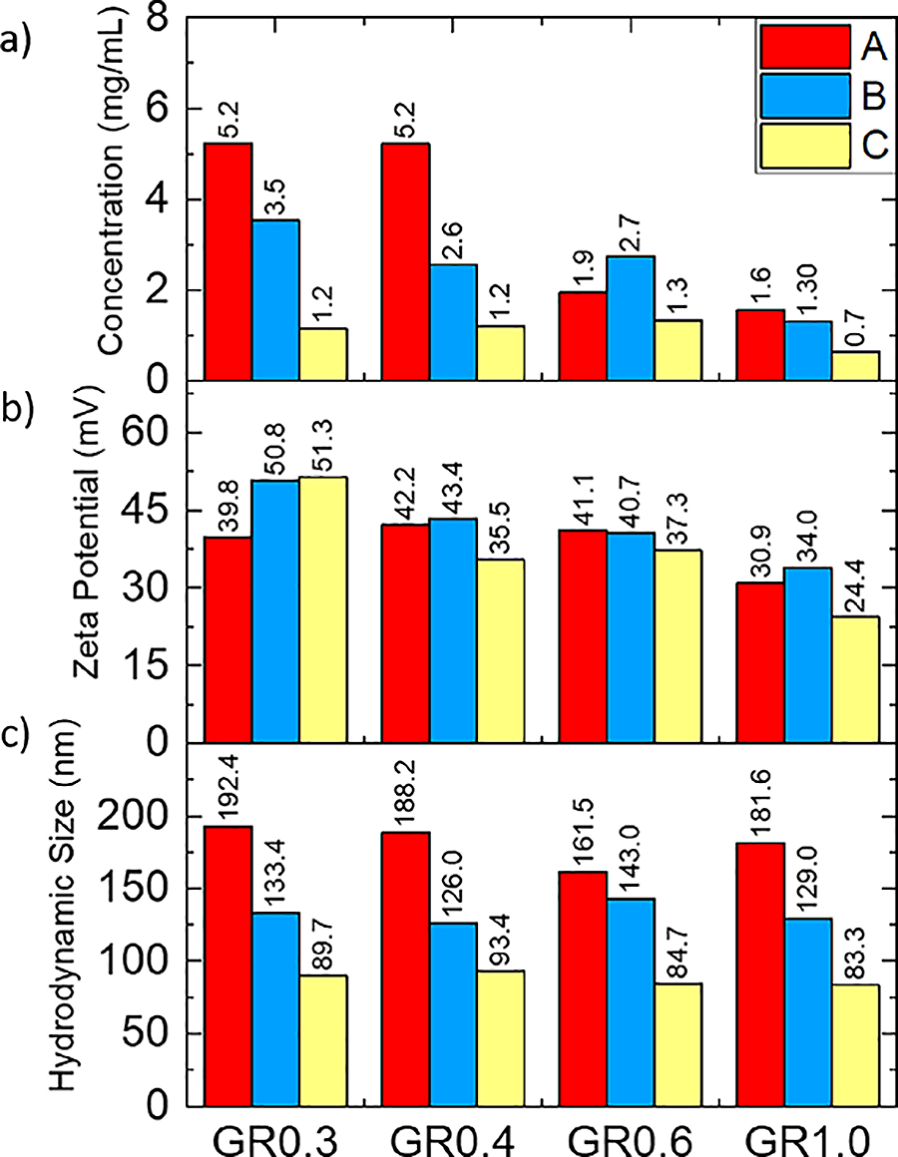
(a) Concentration calculated using the Beer-Lambert law and UV–vis spectroscopy, (b) zeta potential calculated by conversion from μ using Henry’s law and DLS measurements, and (c) hydrodynamic size from DLS measurements of the A, B, and C fractions obtained by LCC made with different initial BPS stabilizer amounts (0.3, 0.4, 0.6, and 1 mg/mL).

To gain understanding of the exfoliation efficiency and stability of each graphene fraction, the concentration, zeta potential, and hydrodynamic size of each fraction were measured (Figure 2). Note that the concentration and stability are in particular very important to perform accurate biological studies.^15^

Figure 2A shows that the concentration of dispersed material reduces with each subsequent fraction. In all samples, however, the concentration remains above 0.5 mg/mL, hence enabling further biological characterization (Section 3.2). Fractions A of the GR0.3 and GR0.4 dispersions are extremely concentrated at ∼5.2 mg/mL, which is significantly higher than the corresponding GR0.6A and GR1.0A fractions, which have concentrations of 1.9 and 1.6 mg/mL, respectively. We note that the dispersion concentration of fraction B roughly decreases with increasing initial BPS concentration, with the GR0.3 having the highest concentration, GR0.4 and GR0.6 having comparable but lower concentrations, and GR1.0 having the lowest. The concentration of fraction C is roughly similar for GR0.3, GR0.4, and GR0.6, whereas the concentration of the GR1.0 fraction is significantly lower. These results may suggest that a lower initial concentration of the BPS stabilizer is favorable to produce highly concentrated dispersions. This facilitates the isolation of fractions containing higher concentrations of smaller and thinner flakes from the stock dispersion, i.e., the higher the concentration of the stock dispersion, the higher the concentration of smaller and thinner flakes within the stock dispersion.

Figure 2B shows the recorded zeta potential of the three fractions from each dispersion: besides fraction C of GR1.0, the zeta potential is always higher than 30 mV, which is the threshold (in absolute value) above which a graphene colloidal suspension is considered stable.^32^ We note a similar rough decreasing trend of zeta potential with increasing initial BPS concentration, which may indicate that the excess stabilizer has a detrimental effect on the dispersion stability.

Figure 2C shows the hydrodynamic size of each fraction obtained by DLS, revealing a clear trend of decreasing hydrodynamic size with each subsequent fraction obtained by centrifugation at higher forces. Additionally, we note that the hydrodynamic size of each fraction is roughly similar regardless of the initial BPS concentration. Although DLS provides only qualitative results, these measurements confirm that the LCC methodology has effectively separated the stock dispersion into fractions with decreasing hydrodynamic size.

TGA was used to analyze the BPS amount in selected graphene dispersions: a higher amount of stabilizer used to prepare the dispersion does not necessarily result in a higher amount of stabilizer adsorbed on the nanosheets as sample preparation involves different steps such as centrifugation and washing, in which molecules may get removed. For simplicity, we selected the GR0.3 and GR0.6 dispersions for TGA analysis. Supporting Information, Figure S1 shows the TGA thermograms for the BPS powder and membranes made from the two selected dispersions. Full details of the thermograms are provided in the Supporting Information. For both graphene dispersions, the mass starts to decrease after 200 °C. There is then a steady decrease in mass until 500 °C where the profiles level off up to the final temperature of 800 °C. We can see that the loss of mass due to the BPS is much higher in the GR0.6 sample as compared to the GR0.3, with the total mass lost for the GR0.6 sample being 32.3% and the mass lost for the GR0.3 sample being 23.4%, indicating a higher amount of BPS in the former sample. It should be noted here that due to the TGA experiments being performed under a nitrogen atmosphere, little-to-no mass loss can be attributed to the loss of carbon from graphene. Hence, in the case of BPS, a higher amount of stabilizer used during exfoliation translates to a higher number of molecules adsorbed on the nanosheets.

**Figure 3:**
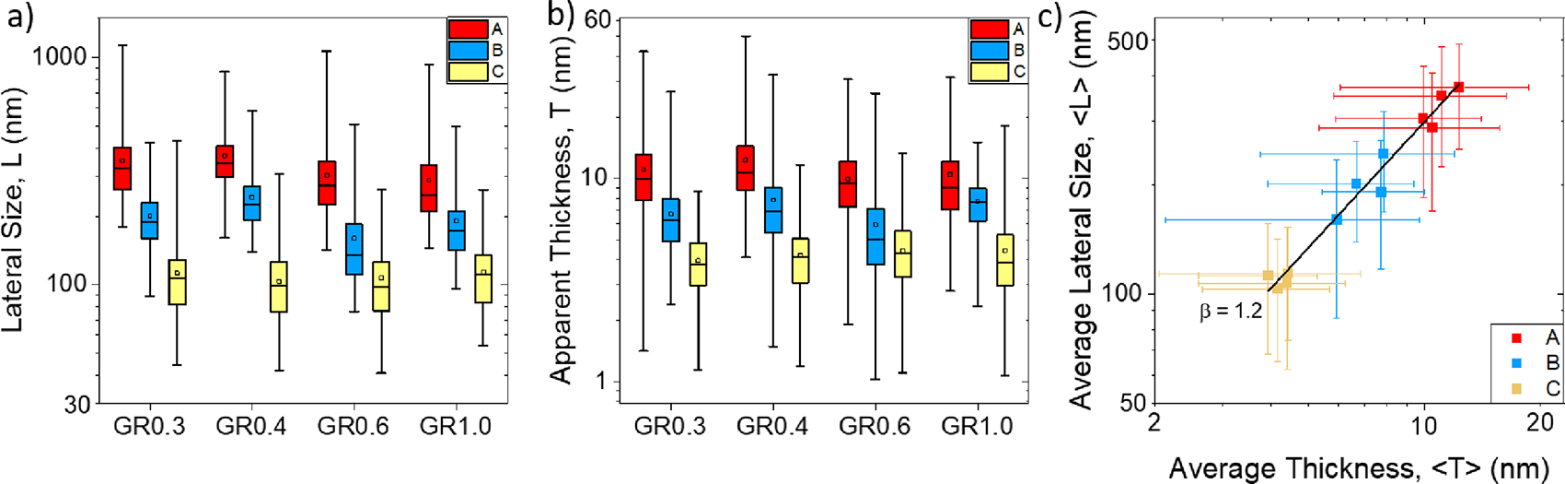
Distributions of (a) lateral size and (b) apparent thickness for GR0.3, GR0.4, GR0.6, and GR1.0 datasets, as measured by AFM. The whiskers show the min/max values, the black square shows the mean, the box shows the interquartile range, and the horizontal line in the box shows the median. (c) Average lateral size vs average thickness for all samples and fractions with error bars showing one standard deviation from the mean and β the gradient of the linear fit. The error bars show the SD in lateral size and apparent thickness.

A statistical AFM methodology^13,33−34^ was then employed to collect a large, representative sample of lateral size and thickness measurements of flakes from fractions from each dispersion, which was then used to evaluate the distribution and to calculate the mean average lateral size (⟨*L*⟩) and mean apparent thickness parameters (⟨*T*⟩), which are reported in the Supporting Information, Table S5. Figure 3A shows the lateral size distributions for each dispersion, which confirms a clear decrease in mean and median lateral size with each subsequent fraction isolated at higher centrifugal forces. For GR0.3, GR0.4, and GR1.0, there is no overlap between the interquartile ranges of each respective fraction, indicating that there is a significant difference between the flake size distributions of each fraction, and hence confirming successful fractioning by size. For sample GR0.6, there is some overlap with the interquartile range of fractions B and C, with the median of each outside the range, which may not only indicate that the fractions are statistically different but also suggests less discrete size selection in this case.

Figure 3B shows the apparent thickness distributions for each dispersion. A clear decrease in mean and median thickness was observed with each subsequent fraction isolated at higher centrifugal forces, in agreement with the trend in lateral size (Figure 3A). There is a very small amount of overlap between the interquartile ranges of fractions A, B, and C of GR0.3 and GR0.4, with the median of each fraction outside the interquartile range of each other. This suggests that there is likely a significant difference between the distributions of flake thicknesses within the two dispersions, and therefore, we have achieved successful fractioning by flake thickness. A larger overlap of interquartile range was observed between fractions for the GR0.6 and GR1.0 dispersions, for GR0.6, particularly between fractions B and C, and for GR1.0, particularly between fractions A and B, with the median of each within the interquartile range of both fractions. This indicates that there is likely no significant difference between the thickness distributions of the two fractions. From each dispersion, fraction B contained flakes mostly <10 nm thick, which is indicative of FLG, whereas the thinnest flakes were typically found in fraction C with the majority of flakes having thickness < 5 nm. The minimum apparent thickness of any flake recorded was ∼1 nm, which is likely indicative of SLG.^19^ In summary, the average flake layer number within the fraction decreases with increased centrifugal force, although the variations are statistically less relevant than those observed with the lateral size. Hence, from our results, we can observe that LCC preferentially sediments and fractionates flakes via lateral size more so than flake thickness.

Figure 3C shows the plot of ⟨*L*⟩ vs ⟨*T*⟩ for each fraction from the four dispersions: this reveals a log-normal trend between size and thickness, which manifests itself as a linear trend on a log–log scale with a gradient of 1.2. This observed trend is in agreement with the results obtained with other pyrene-stabilized graphene dispersions as well as other dispersant-assisted LPE dispersions.^13,33^

We also calculated the ⟨*L*⟩ and ⟨*T*⟩ for the stock dispersion by combining each individual dataset from fractions A, B, and C for each dispersion (Supporting Information, Table S5, sample indicated as “ABC”). This is opposed to taking AFM measurements from samples of the stock dispersion, which would be both challenging and time consuming to do due to the broad range of sizes and thicknesses of flakes within the dispersion. On comparison of the stock dispersion, it appears that the initial BPS concentration has no significant effect on the lateral size or apparent thickness distributions (Supporting Information, Figure S6), with an ⟨*L*⟩ of ∼200 nm and a ⟨*T*⟩ of ∼7 nm recorded for each dispersion.

These results indicate that the liquid cascade used in these experiments has successfully separated the stock dispersion into three discrete fractions by size and thickness, although size-selection by centrifugation appears to be more successful at separating the flakes via size than thickness. However, this may be a result of aggregation and restacking of flakes during sample preparation or from contributions to the overall thickness by residual stabilizer molecules. The stability and concentration of the four dispersions produced and each size-selected fraction were adequate for subsequent biological testing.

**Figure 4:**
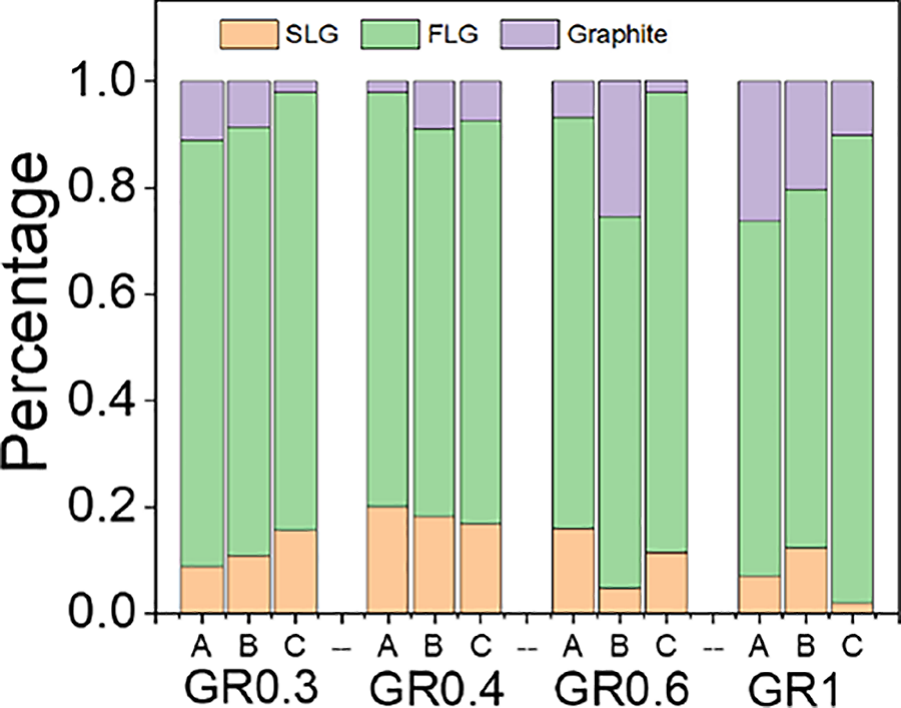
Single-layer graphene (*N* = 1), few-layer graphene (1 > *N* < 7), and graphite (*N* > 10) percentages of each fraction from the four dispersions, extracted from the qualitative analysis of the Raman spectra.

In addition to the AFM measurements, we also employed Raman spectroscopy to characterize over 40 nanosheets from each fraction. The Raman spectrum of the nanosheets shows the characteristic G and D peaks at ∼1580 and ∼1350 cm^–1^, respectively, and the 2D peak at ∼2680 cm^–1^.^36^ The D peak is observed in the spectra of graphene produced by LPE, although in this specific case, the D peak is not attributed to structural defects but it is activated by the edges of the nanosheets, whose size is smaller than the laser spot.^5,19,36,37^ We used a method, developed and tested previously in our group, to qualitatively identify and class the nanosheets as SLG, FLG, or graphite.^15,27−30^Figure 4 shows that all fractions contain between 2 and 20% SLG, 60 and 70% FLG, and 2 and 25% graphite. Tabulated results from Raman spectroscopy can be found in the Supporting Information, Table S7. We note that fraction C typically contains the lowest percentage of graphite and the greatest percentages of SLG and FLG, whereas fractions A and B typically contain higher percentages of graphite due to the LCC process. Additionally, we tend to observe a higher percentage of graphite within the dispersions produced using higher initial BPS concentrations (GR0.6 and GR1.0), which is in agreement with the lowest concentration reported for these samples (Figure 2). These findings are broadly consistent with the AFM data when the apparent thickness was converted into layer number (*N*) using methodology reported in ref (13) (Supporting Information, Table S8 and Figure S9), but the percentage of SLG was significantly lower.

In summary, we applied LCC to the stock dispersions, each produced with a fixed amount of BPS stabilizer, obtaining fractions containing graphene nanosheets with narrow lateral size and thickness distributions. Our results show that a lower initial amount of the BPS stabilizer produces stable dispersions in water with higher concentrations of graphene. By considering the stock dispersion, i.e., combined fractions A, B, and C, we confirm that the initial concentration of the BPS stabilizer has no significant effect on the lateral size or thickness of the nanosheets produced. By comparing results from both AFM and Raman studies, we show that the dispersions contain mostly FLG with <10% graphite in most cases and 10–20% SLG content, which is in agreement with previous results on LPE with pyrene derivatives.^13−15,17^

### 3.2 *In Vitro* Studies

Dimensionality, lateral size, surface charge, and functionalization as well as chemical composition of the nanomaterial can lead to radically different interactions with living systems.^38^ In the framework of 2D materials, graphene oxide (GO) is the most commonly used graphene derivative for biological applications.^39^*In vitro* and *in vivo* effects of GO have been extensively studied.^40^ However, cytotoxicity studies of defect-free graphene obtained by non-covalent functionalization as a function of size and thickness are missing.

**Figure 5:**
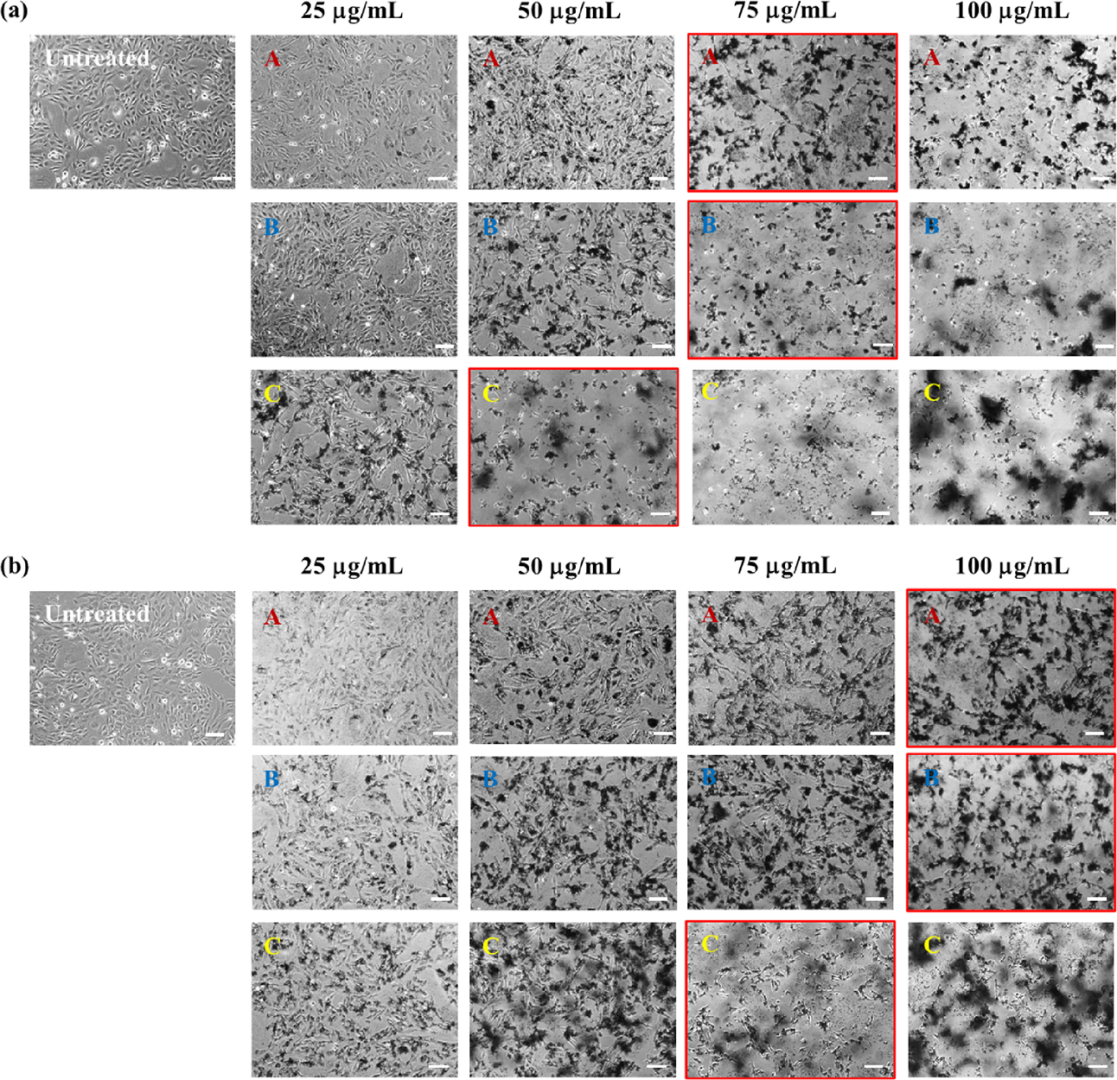
Cytotoxicity of defect-free graphene produced by LPE with BPS in BEAS-2B cells after 24 h of treatment (in the absence of serum for the first 4 h) using different dose concentrations (25, 50, 75, and 100 μg/mL) of (a) GR0.3A/B/C and (b) GR0.4A/B/C, assessed by optical imaging. Images outlined in red indicate the lowest dose concentrations in which cellular stress was observed for each fraction of graphene nanosheets. Scale bars = 100 μm.

**Figure 6:**
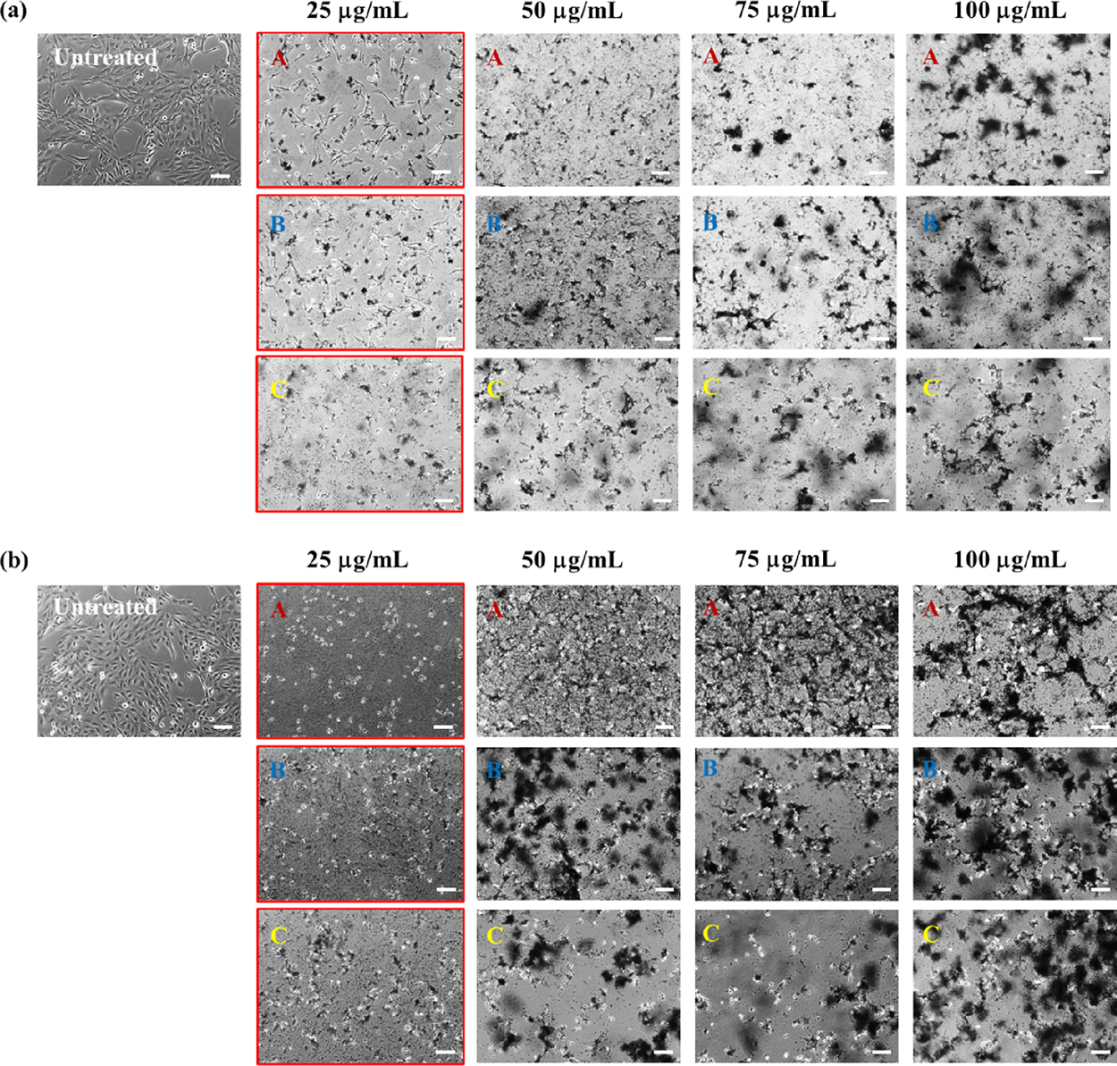
Cytotoxicity of defect-free graphene produced by LPE with BPS in BEAS-2B cells after 24 h of treatment (in the absence of serum for the first 4 h) using different dose concentrations (25, 50, 75, and 100 μg/mL) of (a) GR0.6A/B/C and (b) GR1.0A/B/C, assessed by optical imaging. Images outlined in red indicate the lowest dose concentrations in which cellular stress was observed for each fraction of graphene nanosheets. Scale bars = 100 μm.

The cytotoxicity profile of defect-free graphene produced by LPE with BPS was first assessed via optical imaging of BEAS-2B cells treated with the materials. We remark that cytotoxicity tests on the BPS stabilizer alone cannot be performed as the BPS molecule is insoluble in water.^14^Figures 5 and 6 show optical images of BEAS-2B cells after 24 h of treatment with different concentrations (25, 50, 75, and 100 μg/mL) of graphene sheets exfoliated with lower (GR0.3A/B/C and GR0.4A/B/C) and higher (GR0.6A/B/C and GR1.0A/B/C) amounts of stabilizer. According to the literature, the presence of serum can mitigate the cytotoxicity effect of nanomaterials by lowering the direct contact between the materials and cell membrane. For example, Vranic et al. have found that for both large (5–15 μm) and small (50–200 nm) size GO, a greater cytotoxicity effect is induced in BEAS-2B cells in the absence of FBS.^41^ Herein, for the initial 4 h of exposure, cells were treated with the material without serum to maximize the interaction between graphene sheets and the cells.

It is apparent from Figures 5 and 6 that defect-free graphene prepared using a lower initial amount of stabilizer is less cytotoxic to BEAS-2B cells compared to the ones prepared with higher initial amounts of stabilizer. For example, the lowest dose concentrations in which signs of cellular stress (e.g., reduced cell confluences, cell morphology alteration, and cell detachment) were observed for GR0.3A/B/C and GR0.4A/B/C are 75/75/50 and 100/100/75 μg/mL, respectively. Meanwhile, for GR0.6A/B/C and GR1.0A/B/C, the lowest dose concentration at which cellular stress was observed is at 25 μg/mL.

We also observed increased cellular toxicity with smaller sized graphene. For example, in GR0.3 and GR0.4, the lowest toxic dose concentration is lower for graphene with the smallest size (C) compared to the larger ones (A and B).

**Figure 7:**
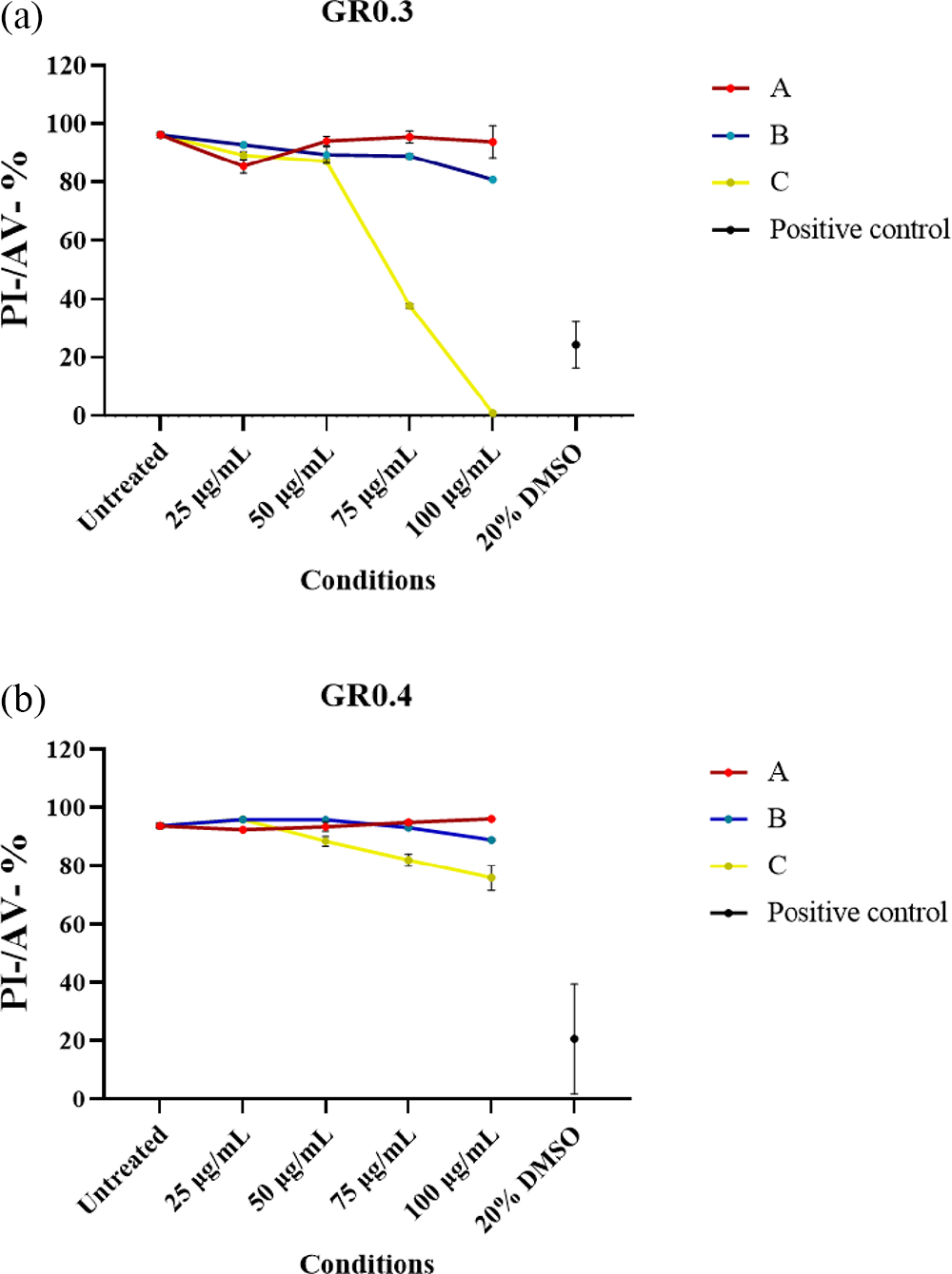
Cytotoxicity of defect-free graphene produced by LPE with BPS in BEAS-2B cells after 24 h of treatment with (a) GR0.3A/B/C and (b) GR0.4A/B/C (25, 50, 75, and 100 μg/mL), assessed by PI/AV staining using flow cytometry. The PI/AV double negative stained cell percentages (PI-/AV- %) were reported. DMSO (20%) was used as the positive control.

To validate the size-dependent effect of defect-free graphene produced by LPE with BPS, the cytotoxicity of GR0.3 and GR0.4 has been further assessed by PI/AV staining using flow cytometry (Figure 7). In agreement with the optical images, the result confirmed the size-dependent toxicity effect of GR0.3 and GR0.4, with smaller size graphene nanosheets causing greater cytotoxicity. For example, in Figure 7, we show that at the highest tested concentration of 100 μg/mL, cell viability (indicated by PI/AV double negative stained cells) reached 93.88% (96.01%) for GR0.3A (GR0.4A), decreased to 80.87% (88.86%) for GR0.3B (GR0.4B), and further lowered to 0.82% (75.81%) for GR0.3C (GR0.4C). It is necessary to clarify that the lowest toxic dose concentration reported using optical imaging refers to the lowest dose concentration at which cellular stress is observed. However, cellular stress does not equate to cell death, hence the discrepancy between visual inspection of cells by optical imaging and percentages of alive cells quantified by flow cytometry.

**Figure 8:**
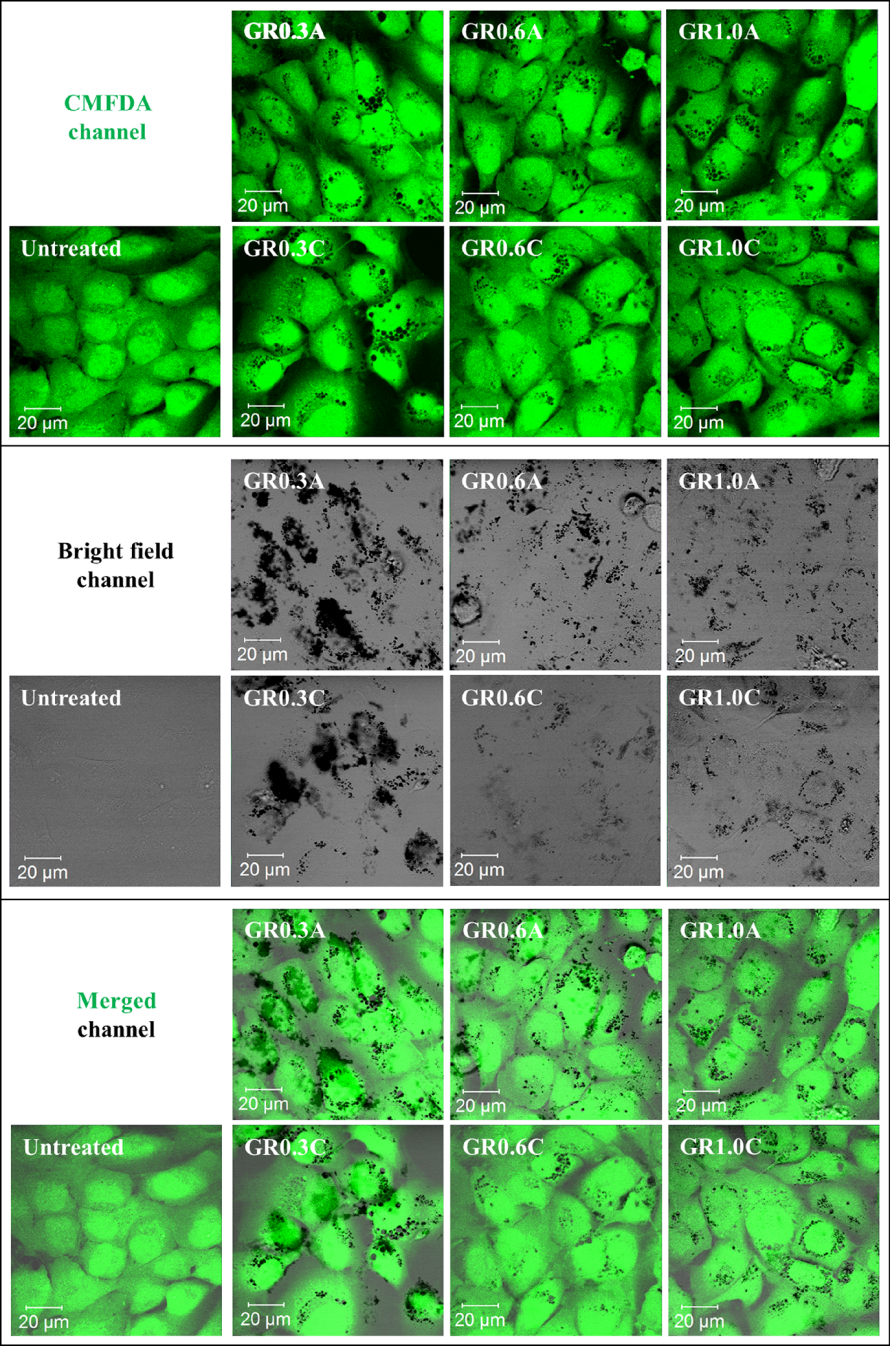
Uptake profile of GR0.3A/C (25 μg/mL), GR0.6A/C (10 μg/mL), and GR1.0A/C (10 μg/mL) in BEAS-2B cells, assessed by confocal imaging (middle section of z-stacks shown). The top, middle, and bottom panels showed the CMFDA, bright field, and merged channel, respectively. See Supporting Information, Figure S10 for images at higher magnifications. Green = CMFDA dye labeled cells, black = graphene flakes.

To understand the potential underlying mechanism of the toxicity induced by the material, the uptake profile for the three sets of graphene sheets (GR0.3, GR0.6, and GR1.0) was assessed in BEAS-2B cells by confocal imaging (see Figure 8). The uptake experiment is performed at sub-toxic dose concentrations (25 μg/mL for GR0.3, 10 μg/mL for GR0.6 and GR1.0). Furthermore, fraction B has been excluded in this experiment to be able to compare the graphene sheets with the largest (A) and smallest size (C). As shown in Figure 8, uptake of GR0.3A/C, GR0.6A/C, and GR1.0A/C was observed in BEAS-2B cells. In general, no obvious differences in the uptake amounts were observed between graphene fractions A and C. BEAS-2B cells are labeled by the CMFDA dye, and in the presence of graphene internalization, the green intracellular fluorescence signal is quenched by the black material. Co-localization of the material in the bright field channel and quenched signal in the CMFDA channel indicates graphene internalization, as clearly shown in Figure 8. The uptake of graphene sheets suggests that the mechanism of cytotoxicity could have been triggered intracellularly. In fact, along with membrane interaction, cellular uptake and intracellular fate of the nanomaterials represent the most common route for initiation of cytotoxicity at the cellular level.^42^

Closer inspection of BEAS-2B cells in all material-treated conditions showed the presence of intracellular vacuoles, mostly found with residing material inside (see Figure 8 and the Supporting Information, Figure S10). This feature is not observed in our previous study on the uptake of different pyrene derivative-stabilized graphene in BEAS-2B cells, and those materials showed exceptional biocompatibility after internalization by the cells.^15^

It has been widely accepted that positively charged nanoparticles are efficiently taken up by the cells. However, they were also found to subsequently cause lysosomal membrane permeabilization (LMP), leading to cell death. In the case of the cationic polystyrene nanosphere, for instance, a “proton sponge” theory has been proposed to explain lysosomal rupture, in which the high proton buffering capacity of the particle surface amines results in excessive proton pump activity and osmotic swelling.^43^ Other cationic nanoparticles, such as cationic polyamidoamine dendrimers, have also been shown to induce LMP as well as loss of mitochondrial membrane potential and apoptosis, with a similar proton sponge mechanism proposed.^44^ Furthermore, comparable features of autophagosome-like vacuoles are shown for BEAS-2B cells treated with single-walled carbon nanotubes that triggered autophagic cell death.^45^ Autophagy is a fundamental catabolic process essential to cell hemostasis maintenance; it can be triggered by physical stress and regulates the degradation of damaged organelles.^46^ The uptake of BPS-stabilized graphene nanosheets and the presence of dense vacuoles hence provide some tentative ideas on the cytotoxic mechanism induced by the material. However, further work is needed to elucidate the mechanism of cytotoxicity stimulated by the BPS-stabilized graphene nanosheets. Future research should investigate whether the materials have triggered lysosomal rupture or autophagy.

Overall, a general size-dependent cytotoxicity effect of BPS-stabilized graphene was found at lower stabilizer concentrations. As shown by optical imaging and the PI/AV assay, the smaller graphene nanosheets appear to be more toxic than the larger ones. The size of the nanomaterial is a well-acknowledged factor that plays a critical role in its resultant toxicity effect. However, contradictory observation as to whether smaller or larger graphene-based nanomaterials are more toxic is seen in the literature,^47^ which is expected due to the different preparation routes and use of different stabilizers. In the case of our defect-free graphene, the initial amount of the stabilizer is found to be the more dominant factor in affecting the cytotoxicity of the material than its size. This is caused by the accumulation of material in intracellular vacuoles, causing lysosomal rupture. As one would expect the amount of adsorbed BPS to increase with the size of the flakes, our results show that the cytotoxicity should not only be related on how much stabilizer is adsorbed but also on how the BPS molecules are adsorbed on the flake as their molecular arrangement can change with increasing stabilizer concentration.^14^

## 4 Conclusions

We demonstrated successful fractioning by LCC of defect-free graphene dispersions in water produced by using the BPS stabilizer. The cytotoxicity profile of the fractions was investigated, showing that the initial amount of the stabilizer is crucial in determining the critical dose at which cellular stress is observed. In particular, for a stabilizer concentration above 0.4 mg/mL, the cytotoxicity does not depend on lateral size or thickness as all fractions show the same critical dose at which cellular stress is observed. As the stabilizer is not soluble in water, this suggests that the cytotoxicity is caused by an excess of molecules adsorbed on the nanosheets. As the cytotoxicity profile changes with the initial amount of stabilizer, this also suggests a different molecular arrangement on the nanosheet above a critical stabilizer concentration. We also observe a strong decrease in the concentration of dispersed graphene and a reduction in zeta potential when using a large amount of stabilizer, further confirming a different molecular arrangement of the excess BPS molecules, which is somehow detrimental to material exfoliation and colloidal stabilization as well as to the cytotoxicity. In contrast, when the stabilizer concentration is below 0.4 mg/mL, the critical dose at which cellular stress is observed depends on the average size of the nanosheet, with the largest nanosheets (average lateral size >200 nm) showing a higher critical dose in comparison to the smaller nanosheets.

## Supporting Information Available

TGA profiles, representative AFM images, tabulated AFM and Raman data, and uptake profiles (PDF)
