# Supplementary material for: Effects of Lateral Size, Thickness, and Stabilizer Concentration on the Cytotoxicity of Defect-Free Graphene Nanosheets: Implications for Biological Applications
Source: ACS Appl Nano Mater. 2022 Sep 12;5(9):12626–36. doi: 10.1021/acsanm.2c02403 (PMC9513747; doi:10.1021/acsanm.2c02403)
Supplement: Supplementary file 1 — an2c02403_si_001.pdf [file an2c02403_si_001.pdf]

Supporting Information

Effect of Lateral Size, Thickness, and Stabiliser Concentration on the Cytotoxicity of Defect-Free Graphene Nanosheets: Implications for Biological Applications

Chen-Xia Hu,[1+] Oliver Read,[1+] Yuyoung Shin,[1] Ying-Xian Chen,[2] Jingjing Wang,[1] Matthew Boyes,[1] Niting Zeng,[1] Adyasha Panigrahi,[1] Kostas Kostarelos,[2,3] Igor Larrosa,[1] Sandra Vranic,[2] Cinzia Casiraghi[1]**

[1]Department of Chemistry, University of Manchester, Oxford Road, Manchester, UK

[2]Nanomedicine Lab, Faculty of Biology, Medicine and Health, AV Hill Building, University of Manchester, Manchester, UK

[3]National Graphene Institute, University of Manchester, Booth Street East, Manchester, UK

*Corresponding authors.

E-mail addresses: cinzia.casiraghi@manchester.ac.uk, sandra.vranic@manchester.ac.uk

Content

S1. TGA Profile

S2. Representative AFM Images

S3. Lateral Size / Thickness Aspect Ratio

S4. Tabulated AFM Data

S5. Stock Dispersion ('ABC') Lateral Size and Thickness Distributions

S6. Tabulated Raman Data

S7. Tabulated AFM Layer Number Data

S8. Raman vs AFM-Layer Number percentages

S9. Uptake Profile

S1. TGA Profile

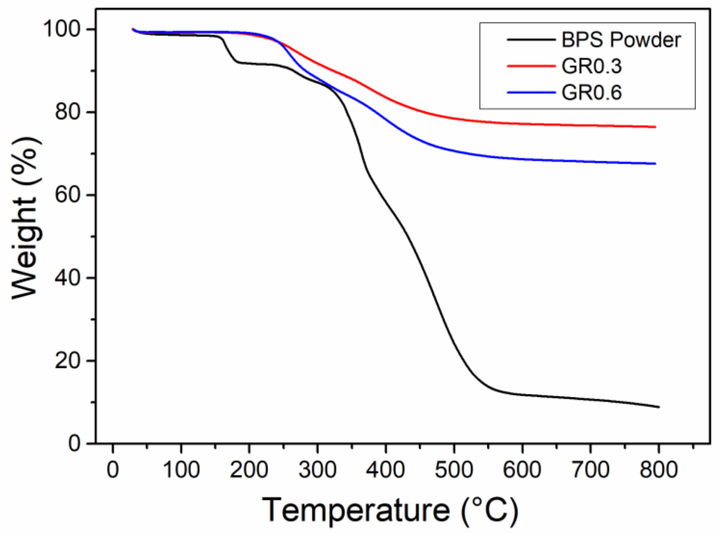

Figure S1.1: TGA thermograms for the BPS powder (black trace) and membranes made from graphene dispersions prepared using initial BPS concentrations 0.3 and 0.6 mg/mL (red and blue traces, respectively).

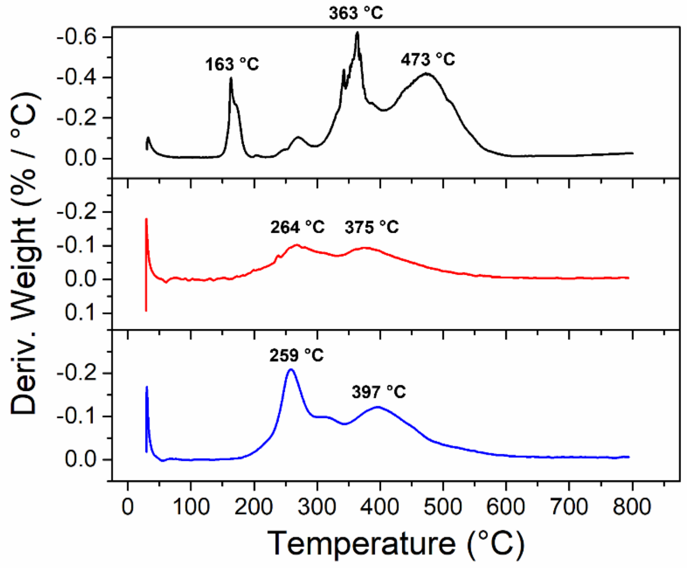

Figure S1.2: Traces of the first derivative with respect to *T* of the TGA thermograms for the BPS powder (black trace) and membranes made from graphene dispersions prepared using initial BPS concentrations 0.3 and 0.6 mg/mL (red and blue traces, respectively).

Figure S1.1 details the TGA thermograms of the pure BPS powder and both graphene dispersions analysed. All thermograms show a small initial mass loss below 50 °C, which is likely to be trace amounts of water. Examining the thermogram for the pure powder reveals

that nearly all the material is lost at 800 °C, with presumably the remaining 10% weight being graphitic carbon residue that can not be burned in an inert atmosphere. The thermograms of the graphene dispersions show much less overall weight loss compared to the pure powder. The much higher amount of carbon in these samples because of the graphene means that less of the material can be lost upon heating.

The derivatives of the thermograms from Figure S1.1 gives the results shown in Figure S1.2. This process allows for facile identification of mass loss events, and the respective rate of loss, at a given temperature. Given the scope of the research undertaken in this work, it is impossible to say with certainty what the mass loss events mean with regards to sample degradation. Hence, investigating these uncertainties, as well as the impact of long sonication times on the BPS molecule are further points of interest for future work. However, some clear observations can be made from the data that has been acquired. The pure BPS powder has seemingly 3 distinct mass loss events: a rapid loss at 163 °C, another rapid loss at 363 °C, and a final relatively slow loss at 473 °C. The traces for the graphene dispersions are very different to the pure BPS powder. First of all, they do not show the initial rapid loss at 163 °C. With the evidence collected, this is likely attributed to the preparation method of the graphene dispersions for TGA where the material was subjected to drying temperatures of 250 °C under vacuum, so the loss of mass at 163 °C already occurred. Secondly, both graphene materials show two separate mass loss events but at significantly lower temperatures than the pure BPS powder, suggesting a lower thermal stability of BPS when it is adsorbed onto graphene nanosheets. This is contradictory to conventional understanding of adsorbed molecules on graphene nanosheets[1] and reinforces the desire for further work.

S2. Representative AFM Images

<u>GR0.3</u>

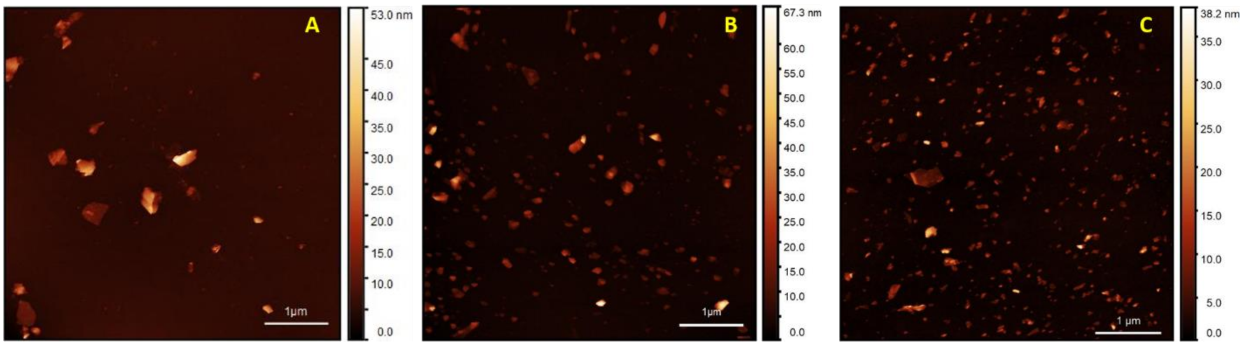

<u>GR0.4</u>

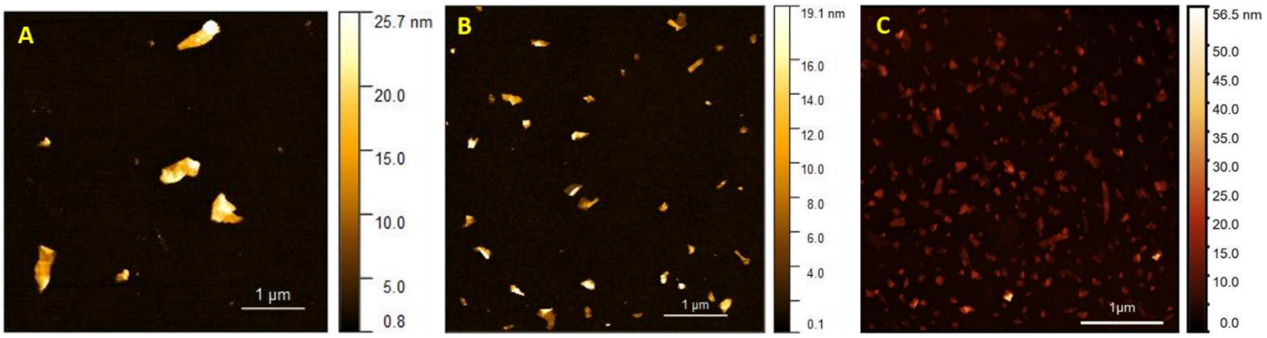

<u>GR0.6</u>

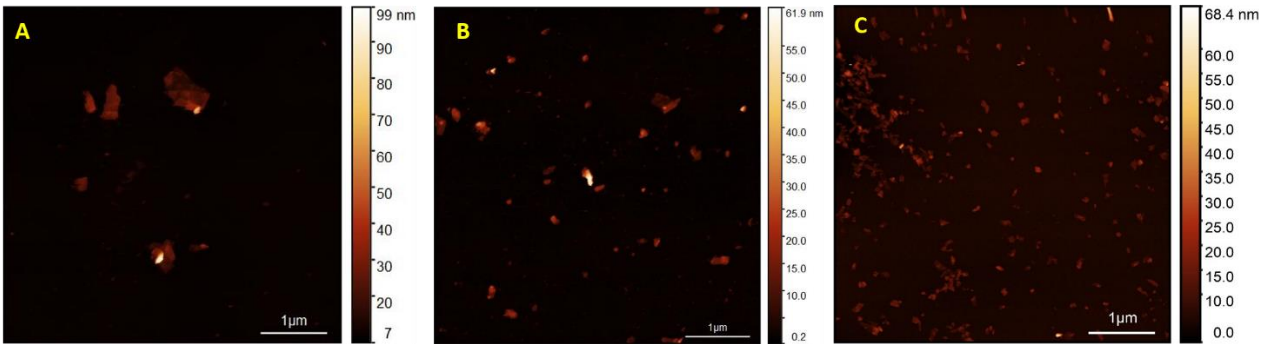

<u>GR1.0</u>

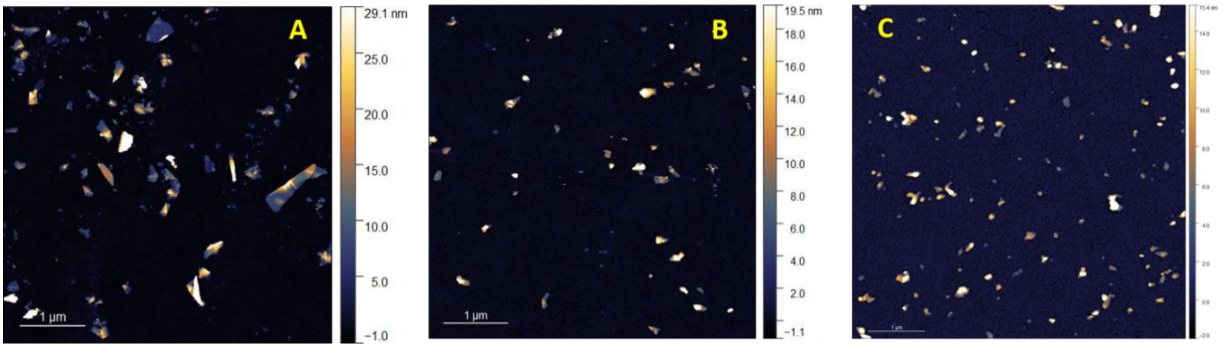

Figure S2: Representative AFM images of fractions A, B and C for BPS-stabilised graphene dispersions produced using 0.3, 0.4, 0.6 and 1.0 mg/mL of BPS.

S3. Lateral Size / Thickness Aspect Ratio

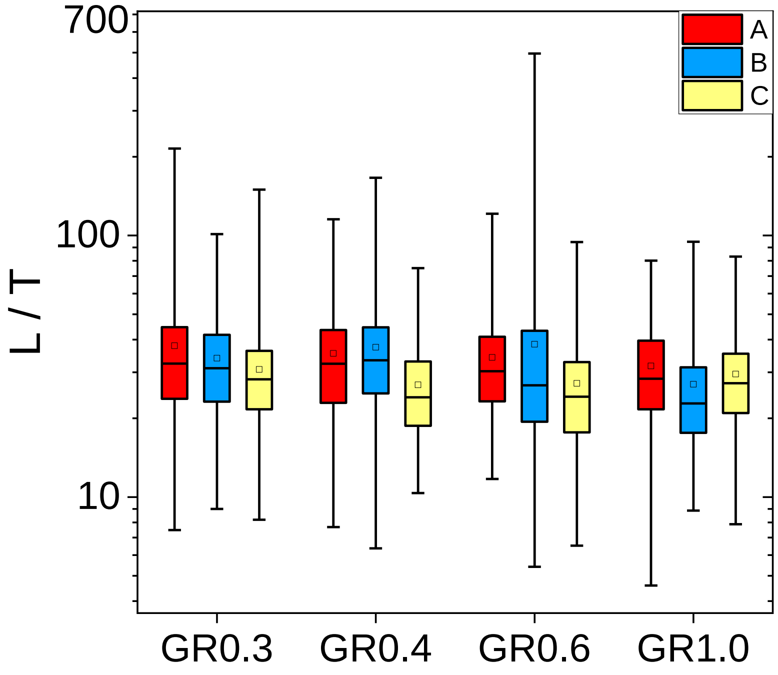

Figure S3: Distributions of the lateral size / thickness aspect ratio (L/T) for GR0.3, GR0.4, GR0.6 and GR1.0 data sets, as measured from AFM. The whiskers show the min/max values, the black square the mean, the box the interquartile range and the horizontal line in the box the median.

S4. Tabulated AFM Data

Table S4. Mean average lateral size and mean average apparent thickness for each fraction of the four BPS-stabilised graphene dispersions at differing initial amount of BPS. Also included are the values for the stock dispersion, i.e. the combined fractions labelled 'ABC'. Each value is quoted with one standard deviation.

Dispersion	Fraction	<L> / nm	<T> / nm	<L/T>	Flake Count
GR0.3	A	352.1 ± 127.2	11.1 ± 5.3	37.9 ± 23.6	349
	B	201.5 ± 62.3	6.7 ± 2.7	33.9 ± 14.7	368
	C	112.4 ± 44.1	3.9 ± 1.3	30.8 ± 13.6	824
	ABC	187.9 ± 121.8	6.2 ± 4.1	33.1 ± 16.8	1541
GR0.4	A	370.5 ± 119.1	12.3 ± 6.3	35.4 ± 17.9	197
	B	243.2 ± 74.8	7.8 ± 4.1	37.4 ± 20.8	334
	C	103.4 ± 38.3	4.2 ± 1.5	26.8 ± 11.2	496
	ABC	200.1 ± 126.2	6.9 ± 4.9	31.9 ± 16.9	1027
GR0.6	A	304.5 ± 120.3	9.9 ± 4.1	34.2 ± 15.8	473
	B	160.0 ± 74.1	5.9 ± 3.8	38.4 ± 45.7	145
	C	107.2 ± 45.3	4.4 ± 1.8	27.2 ± 13.3	206
	ABC	229.8 ± 132.7	7.9 ± 4.4	33.2 ± 23.8	824
GR1.0	A	287.8 ± 118.0	10.5 ± 5.1	31.7 ± 14.7	170
	B	191.2 ± 73.6	7.7 ± 2.3	27.0 ± 13.6	150
	C	113.8 ± 39.3	4.4 ± 2.4	29.5 ± 12.3	184
	ABC	195.5 ± 110.3	7.5 ± 4.4	29.5 ± 13.6	503

S5. Stock Dispersion ('ABC') lateral Size and thickness distributions

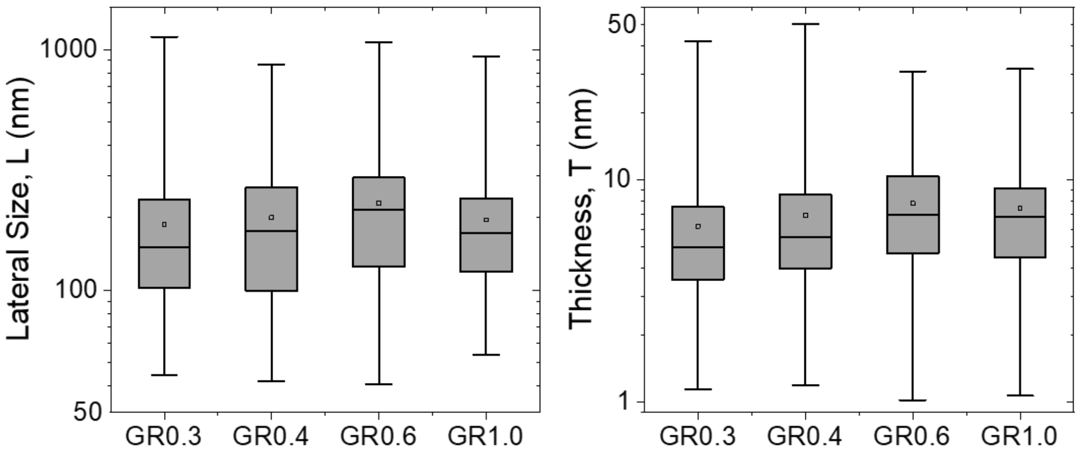

Figure S5. Boxplots of lateral size (nm) and thickness (nm) for each stock dispersion (i.e. combined fractions 'ABC') of BPS stabilised graphene at differing initial amounts of BPS. The black square shows the mean, the box the interquartile range with the horizontal line in the box indicating the median, and the whiskers showing the min/max values.

We show that there is no significant difference in lateral size or thickness distributions of flakes within each of the stock graphene dispersions, due to the overlapping interquartile ranges of each distribution. Hence, we show that the initial concentration of BPS stabiliser has no effect on the size or thickness distribution of flakes, similarly to previous reports in which the type of pyrene stabiliser had no effect on the size or thickness distributions.[2]

S6. Tabulated Raman Data

Table S6. Population and relative percentages of graphene flake layer number (N) from fractions of each dispersion as determined by Raman Spectroscopy. Where SLG = single layer graphene (N=1), FLG = few layer graphene (N = 2-7) and Bulk = graphite (N>10).

Sample		Fraction					
		A		B		C	
**Gr0.3**	**SLG**	3	8.60%	3	8.30%	7	15.60%
	**FLG**	28	80.00%	30	83.30%	37	82.20%
	**Bulk**	4	11.40%	3	8.30%	1	2.20%
	#	**35**	**100.00%**	**36**	**100.00%**	**45**	**100.00%**
**Gr0.4**	**SLG**	8	23.50%	8	18.20%	9	16.70%
	**FLG**	25	73.50%	32	72.70%	41	75.90%
	**Bulk**	1	2.90%	4	9.10%	4	7.40%
	#	**34**	**100.00%**	**44**	**100.00%**	**54**	**100.00%**
**Gr0.6**	**SLG**	7	15.90%	2	4.70%	5	11.40%
	**FLG**	34	77.30%	30	69.80%	38	86.40%
	**Bulk**	3	6.80%	11	25.60%	1	2.30%
	#	**44**	**100.00%**	**43**	**100.00%**	**44**	**100.00%**
**Gr1.0**	**SLG**	3	7.10%	6	12.30%	1	2.00%
	**FLF**	28	66.70%	33	67.30%	43	87.80%
	**Bulk**	11	26.20%	10	20.40%	5	10.20%
	#	**42**	**100.00%**	**49**	**100.00%**	**49**	**100.00%**

S7. Tabulated AFM Layer Number Data

Table S7. Population and relative percentages of graphene flake layer number (N) from fractions of each dispersion as determined by atomic force microscopy using methodology outlined in previous work.[2] Where SLG = single layer graphene (N=1), FLG = few layer graphene (N = 2-10) and Bulk = graphite (N>10).

Sample		Fraction					
		A		B		C	
Gr0.3	SLG	0	0%	0	0%	1	0.12%
	FLG	69	19.77%	248	67.40%	806	97.82%
	Bulk	280	80.23%	120	32.60%	17	2.06%
	#	**349**	**100.00%**	**368**	**100.00%**	**824**	**100.00%**
Gr0.4	SLG	0	0%	0	0%	2	0.40%
	FLG	23	11.68%	188	56.29%	478	96.37%
	Bulk	174	88.32%	146	43.71%	16	3.23%
	#	**197**	**100.00%**	**334**	**100.00%**	**496**	**100.00%**
Gr0.6	SLG	0	0%	4	2.76%	6	2.91%
	FLG	122	25.79%	109	75.17%	191	92.72%
	Bulk	351	74.21%	32	22.07%	9	4.37%
	#	**473**	**100.00%**	**145**	**100.00%**	**206**	**100.00%**
Gr1.0	SLG	0	0%	0	0%	1	0.54%
	FLG	44	26.04%	67	44.67%	166	90.22%
	Bulk	125	73.96%	83	55.33%	17	9.24%
	#	**169**	**100.00%**	**150**	**100.00%**	**184**	**100.00%**

S8. Raman vs AFM-Layer Number percentages

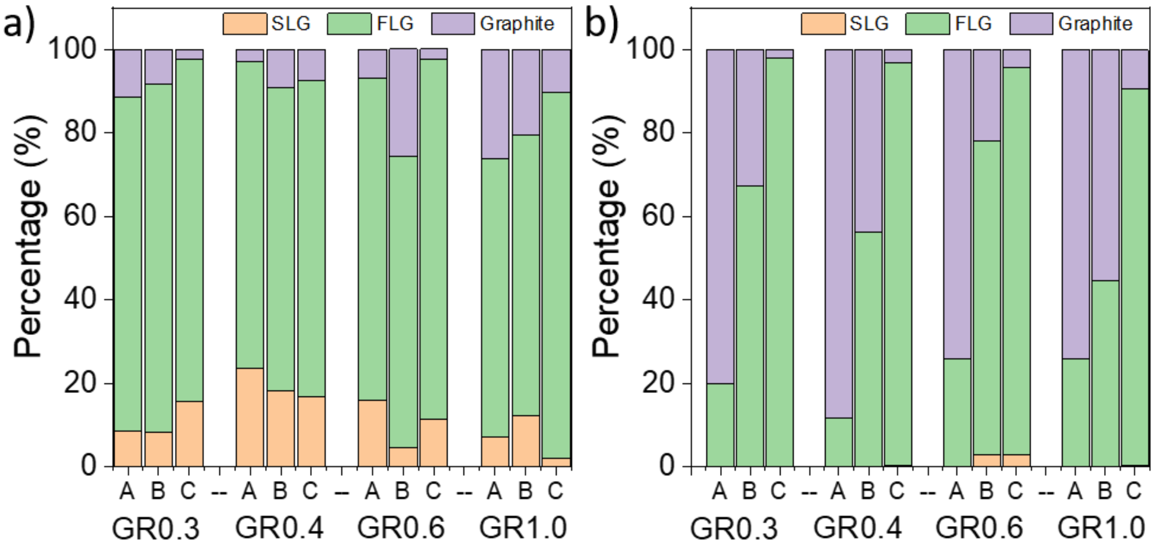

Figure S8. Relative percentages of SLG, FLG and bulk graphite flakes as determined by a) Raman Spectroscopy and b) Atomic Force Microscopy.

The apparent thickness of each nanosheet as measured by AFM was converted to N following methodology in previous work.[2] A clear decrease in bulk graphite percentage and increase in FLG percentage was seen with increasing centrifugal force from fractions A to C for each sample. SLG percentage is very low in all cases, when compared to the results from Raman, with only a few single layer flakes identified over the entire data set. This is likely to be because of the inherent issues faced when measuring the thickness of solution-processed nanosheets due to residual stabiliser and solvent molecules above and below the nanosheets contributing to the overall thickness. Hence, there are also issues with estimating N from AFM apparent thickness when using stabiliser molecules which can lead to vast over or underestimations of N depending on which method is used. The results from AFM are therefore only qualitative and allow us to see that generally, flakes reduce in thickness and therefore layer number with increased centrifugal force, which is in agreement with the layer number data from Raman Spectroscopy.

S9. Uptake Profile

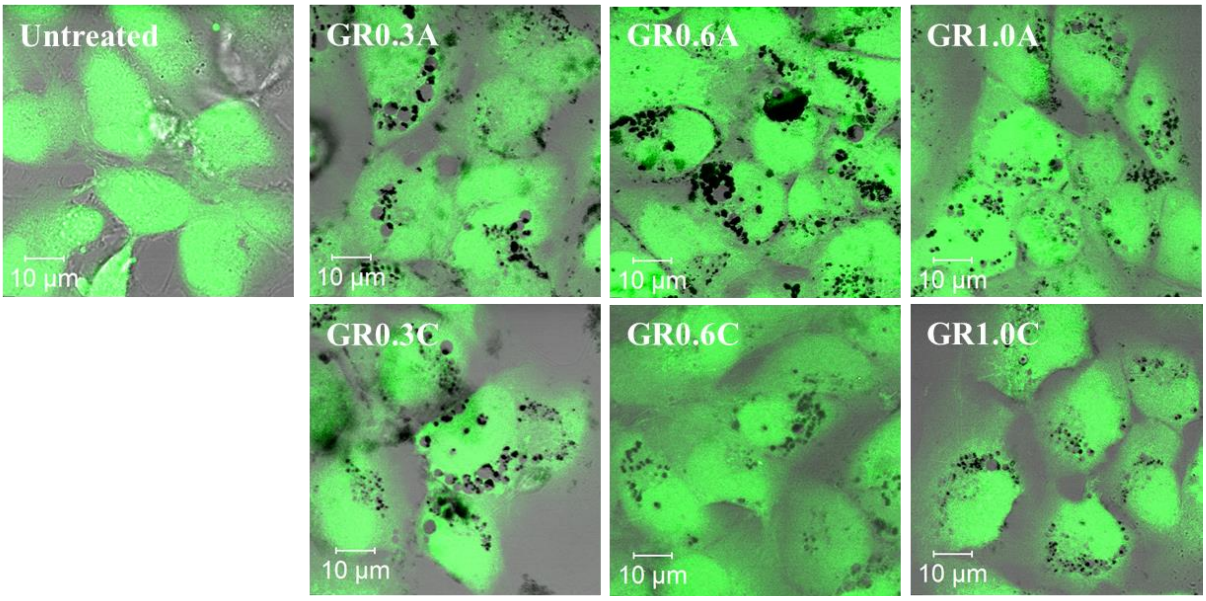

Figure S9. Uptake profile of GR0.3A/C (25 µg/mL), GR0.6A/C (10 µg/mL) and GR1.0A/C (10 µg/mL) in BEAS-2B cells by confocal imaging (middle section of z-stacks shown). See **Figure 8** for images at lower magnifications. Green = CMFDA dye labelled cells, black = graphene flake.

Bibliography

1 A. M. Díez-Pascual, C. Vallés, R. Mateos, S. Vera-López, I. A. Kinloch and M. P. S. Andrés, Influence of surfactants of different nature and chain length on the morphology, thermal stability and sheet resistance of graphene, *Soft Matter*. 2018, **14**, 6013–6023.

2 O. Read, Y. Shin, C. Hu, M. Zarattini, M. Boyes, X. Just-Baringo, A. Panigrahi, I. Larrosa and C. Casiraghi, Insights into the exfoliation mechanism of pyrene-assisted liquid phase exfoliation of graphene from lateral size-thickness characterisation, *Carbon* 2021, **186**, 550–559.
